# The Genome of the Stick Insect *Medauroidea extradentata* Is Strongly Methylated within Genes and Repetitive DNA

**DOI:** 10.1371/journal.pone.0007223

**Published:** 2009-09-29

**Authors:** Veiko Krauss, Carina Eisenhardt, Tina Unger

**Affiliations:** Department of Genetics, Institute of Biology II, University of Leipzig, Leipzig, Germany; University of Munich and Center of Integrated Protein Science, Germany

## Abstract

**Background:**

Cytosine DNA methylation has been detected in many eukaryotic organisms and has been shown to play an important role in development and disease of vertebrates including humans. Molecularly, DNA methylation appears to be involved in the suppression of initiation or of elongation of transcription. Resulting organismal functions are suggested to be the regulation of gene silencing, the suppression of transposon activity and the suppression of initiation of transcription within genes. However, some data concerning the distribution of methylcytosine in insect species appear to contradict such roles.

**Principal Findings:**

By comparison of MspI and HpaII restriction patterns in genomic DNA of several insects we show that stick insects (Phasmatodea) have highly methylated genomes. We isolated methylated DNA fragments from the Vietnamese Walking Stick *Medauroidea extradentata* (formerly known as *Baculum extradentatum*) and demonstrated that most of the corresponding sequences are repetitive. Bisulfite sequencing of one of these fragments and of parts of conserved protein-coding genes revealed a methylcytosine content of 12.6%, mostly found at CpG, but also at CpT and CpA dinucleotides. Corresponding depletions of CpG and enrichments of TpG and CpA dinucleotides in some highly conserved protein-coding genes of Medauroidea reach a similar degree as in vertebrates and show that CpG methylation has occurred in the germline of these insects.

**Conclusions:**

Using four different methods, we demonstrate that the genome of *Medauroidea extradentata* is strongly methylated. Both repetitive DNA and coding genes appear to contain high levels of methylcytosines. These results argue for similar functions of DNA methylation in stick insects as those already known for vertebrates.

## Introduction

Variable proportions of cytosine residues in eukaryotic genomes are methylated. The percentage of methylcytosines ranges from 0–10% in insects, about 3–10% in mammals and birds, about 10% in fish and amphibians up to 50% in some plants [1], [2]. DNA methylation has been associated with numerous functions, depending on the model organism and the experimental context. Molecularly, DNA methylation in animals and plants is associated with the inhibition of initiation of transcription [3]. In contrast, some fungi seem to use DNA methylation to inhibit the elongation of transcription [4].

Although there have been many studies on DNA methylation, to establish its primary role that has led to its widespread distribution in higher organisms has proved to be controversial. It might be the suppression of activity of transponible elements [5], [6]. It has also been proposed that DNA methylation primarily acts to stabilize patterns of endogenous gene activity by maintaining gene silencing that was build by other means [7]. This could be used to transmit determined and differentiated states in cell lineages [8]. Third, it was suggested that DNA methylation located inside of transcription units of animals and plants could prevent spurious internal initiation of transcription [9], [10].

To understand DNA methylation, insects appear to be especially revealing. The available data demonstrate widely varying levels of methylation in several insect species belonging to various orders and do not seem to indicate any conserved biological function [2]. At first glance, in some insect species DNA methylation plays only a minor role or appears not to silence genes but to keep them in activity [2]. Methylation of transposons could only be shown for Drosophila [11], but not for other methylated insect genomes [12]. So, insects might represent a group of animals where DNA methylation occurs only spuriously, is distributed differently and has biological functions unrelated to those found in other metazoans. However, this impression may vanish if species would be found that show more familiar patterns of DNA methylation.

To find such species, we compared insects with angiosperms and vertebrates, which show the highest levels of DNA methylation. Both groups contain rather big multicellular organisms with large genomes, a high cell turnover during a long individual lifetime and, correspondingly, relatively low population sizes. Such conditions facilitate cytosine methylation irrespective of the resulting cytosine hypermutability, that is, the especially high rate of mutation of methylcytosine to thymine [13]. On the other hand, DNA methylation is a mutational burden [14] that will be selected against in organisms which have (1) less need for methylation because of relatively short transcription units (correlated with small genome sizes) and a low cell turnover during development [8] and (2) a large population size facilitating selection. These conditions are more or less fulfilled in insects that have already been analyzed for DNA methylation (e.g. fruit flies, mosquitos, butterflies, bees, aphids and mealy bugs), so it is conclusive that strongly methylated genomes could not be found here.

A group of insects with rather large genome sizes – 2 to 8 pg per haploid genome, which come close second to orthopterans – are walking sticks (Phasmatodea) [15]. This group contains the largest species of insects and constitutes an independent lineage since about 300 million years [16]. Walking sticks often reproduce parthenogenetically which should result in especially low effective population sizes as well as in high cell turnover in relation to other insects. They need a long time, typically several months, for development also in tropical habitats. To our knowledge, DNA methylation has not been evaluated yet in these insects. Thus, we decided to analyze the occurrence and distribution of methylcytosine in the Vietnamese Walking Stick, *Medauroidea extradentata*.

By comparison of digestion patterns produced by a pair of restriction enzymes showing different sensitivity against methylcytosine, we demonstrate that two species of walking sticks have a strong, genome-wide CpG methylation. We isolated methylated DNA fragments from *M. extradentata* and learned that most of the corresponding sequences are repetitive. Bisulfite sequencing of one of these fragments and of parts of a conserved protein-coding gene revealed a methylcytosine content of 12.6%, mostly found at CpG, but also at CpT and CpA dinucleotides. In addition, the alignment of orthologous sequences from different genomes demonstrates that the depletion of CpG and the corresponding enrichment of TpG and CpA dinucleotides inside of protein-coding genes reaches a similar degree as in vertebrates and reveals a history of CpG methylation in the germline of these insects. Therefore, we suggest that in stick insects DNA methylation has similar functions as in vertebrates.

## Results

### Detection of methylation by differential digestion

**Figure 1:**
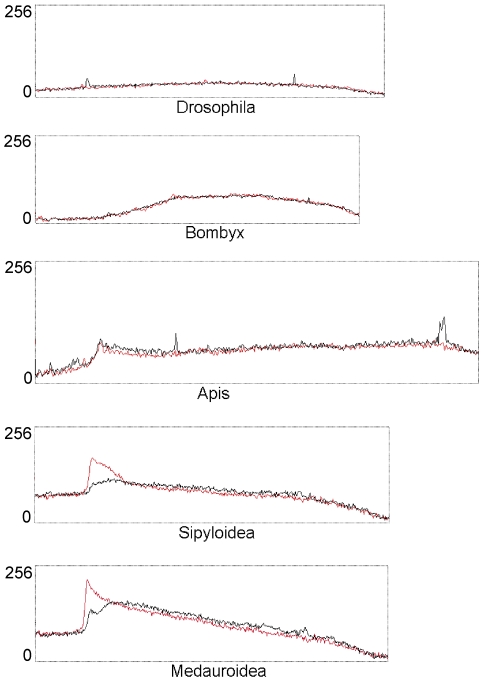
MspI/HpaII restriction analysis of selected insect species. Equivalent amounts of insect genomic DNA were digested with MspI (black line) and HpaII (red line) and separated at adjacent lanes of an agarose gel. The ethidium bromide signals of both lanes were plotted in different colors into one plot. Results are shown for *Drosophila melanogaster, Bombyx mori, Apis mellifera* and the walking sticks *Sipyloidea sipylus* and *Medauroidea extradentata*.

We started our analysis with a comparative digestion of genomic insect DNA using the restriction enzymes MspI and HpaII. Both enzymes are isoschizomers that recognize the target sequence 5′-CCGG-3′, but only HpaII is inhibited by methylation of the inner cytosine of this sequence. If the digestion pattern of HpaII is shifted to higher molecular weights in relation to the MspI pattern, the corresponding genome contains mCpG (methylcytosine, followed by a guanine). The genomic DNA of two walking sticks (*Sipyloidea sipylus* and *Medauroidea extradentata*) clearly showed such a difference (Figure 1), while DNA of *Drosophila melanogaster* (Diptera), *Bombyx mori* (Lepidoptera) and *Apis mellifera* (Hymenoptera) was equally digested by MspI and HpaII. We note that for Apis and Bombyx CpG methylation was shown [17], [18], but the fraction of methylated CpG is probably rather low in both species.

**Figure 2:**
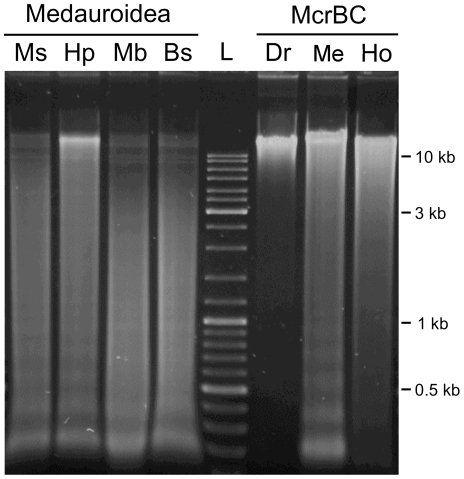
Differential restriction analysis of *Medauroidea extradentata*. Equivalent amounts of genomic DNA were digested and analyzed using MspI (Ms), HpaII (Hp), MboI (Mb), Bsp143I (Bs) and McrBC. For comparison, equivalent genomic digestions using McrBC and DNA from *Drosophila melanogaster* (Dr), *Medauroidea extradentata* (Me) and *Homo sapiens* (Ho) are shown. The track labelled L contains GeneRuler® Ladder Mix (Fermentas).

A second pair of isoenzymes, MboI and Bsp143I, was used to detect a possibly general cytosine methylation irrespective of the nucleotide downstream of methylcytosine. Both enzymes are isoschizomers that recognize the same target sequence 5′-GATC-3′, but only Bsp143I is inhibited by methylation of the cytosine within this sequence [19]. In addition, we used the methylation-specific enzyme McrBC, which recognizes 5′-RmC(N_40–3000_)RmC-3′ sites. We found no obvious differences between the digestion patterns of MboI and Bsp143I, which argues against a significant non-CpG methylation (Figure 2). The McrBC digestion of Medauroidea DNA resulted in an evenly distributed smear and a strong undigested band. In contrast, *Drosophila melanogaster* DNA stayed essentially undigested, and the human DNA was more completely digested. Based on these results, we suggest that *Medauroidea extradentata* has a significant DNA methylation, which is mainly found at CpG sites at some regions of the genome, whereas other regions are only weakly or not methylated.

To isolate methylated fragments of *M. extradentata* DNA, we eluted the DNA peak of the HpaII lane located at the higher molecular weight boundary of the separation area, digested the elution with Bsp143I and cloned the resulting fragments (Materials and Methods). 39 fragments were selected (1) to contain at least one internal HpaII or Bsp143I restriction site and (2) to be larger than 200 bp, and were sequenced. Omitting doublets and bacterial contaminations, we ended up with 23 sequences containing supposedly methylated cytosines in 5′-CCGG-3′ (HpaII) or 5′-GATC-3′ (Bsp143I) restriction sites. According to a BLAST analysis, none of these 23 sequences contains a significantly conserved part of a gene.

**Figure 3:**
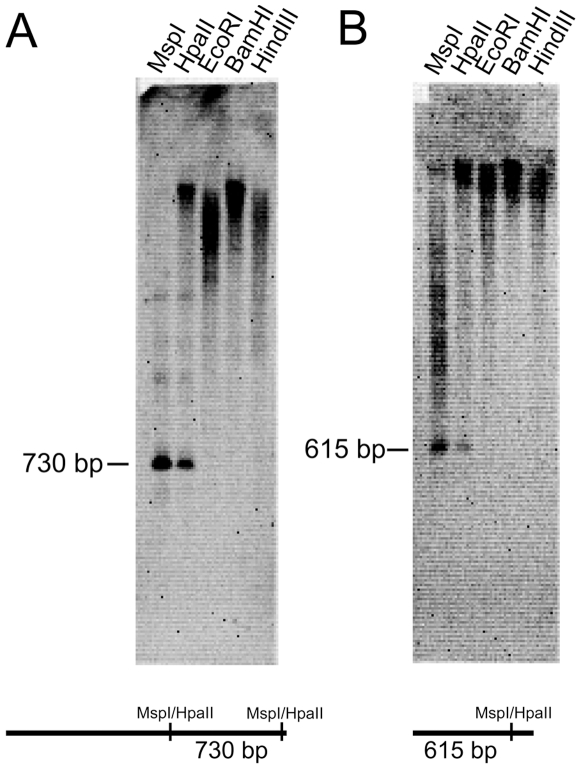
Southern blots showing methylation and repetitivity of selected DNA fragments. Both blots contain five enzyme lanes. CpG methylation is demonstrated by a partial signal shift from lower (MspI lane) to higher molecular weights (HpaII lane). The existence of more than two signals in the lanes may be due to restriction site variability between different copies of the repetitive sequences or due to methylation of the outer cytosine of some MspI sites, which renders such sites resistent to MspI. (A) The 730 bp signal corresponds to the expected internal MspI/HpaII fragment as shown below for the 1862 bp fragment 3–20 (FM985962). (B) The 615 bp signal may correspond to the longer MspI/HpaII fragment of the 835 bp fragment 5–11 (FM985964).

Out of these clones, 10 larger fragments with at least one HpaII site (615 to 2526 bp, accession numbers FM985962-FM985971) were evaluated by Southern blot for methylation of the inner cytosine of 5′-CCGG-3′ and for uniqueness within the Medauroidea genome (for two examples, see Figure 3). All 10 fragments were shown to be, at least in part, methylated. Nine of these 10 fragments are repetitive, as demonstrated by strong and diffuse hybridization signals in EcoRI, BamHI and HindIII lanes (Figure 3). The frequent incompleteness of methylation might be due to (1) differential methylation of different copies in all cells and/or (2) differential methylation of the same genomic copies in different tissues. In summary, 14 out of 23 fragments are repetitive, as shown by Southern Blot (9), internal repeats (6), and/or by BLAST analysis (6). Typical satellite sequences, consisting of multiple tandem repeats, were not identified. Interestingly, best BLAST hits revealed that 4 fragments contained remnants of reverse transcriptase genes. Three of these fragments (5–27, 7–29 and 7–41) were used as Southern probes. All revealed an incomplete methylation of the 5′-CCGG-3′ sites. Two other, not further evaluated fragments correspond, according to BLAST hits, to a DNA transposase gene or to a pseudogene, interrupted by several translation stops, of phospholipase c beta.

### Bisulfite sequencing

Next, we undertook bisulfite sequencing experiments to obtain (1) the abundance of methylcytosine and (2) the nucleotides adjacent to methylcytosines in selected sequences. Sodium bisulfite reverts cytosines to uraciles, but leaves 5mC unchanged. Thus, methylcytosines could be identified by sequencing of bisulfite PCR clones (Materials and Methods). Initially, we used one fragment which has been confirmed to be methylated and contained a remnant of a reverse transcriptase gene (7–29, 2216 bp, accession number FM985968). However, only 4 bisulfite clones could be obtained from this repetitive sequence. Therefore, we additionally analyzed subfragments of three protein-coding genes (for isolation see below). Together, 64 bisulfite clones were sequenced from these genes (Table 1). Including the retrotransposon sequences, we obtained 14616 bp sequence containing 2851 (19.5%) cytosines.

**Table 1:** Bisulfite sequencing.

	Accession number	Bisulfite clones	mCs	mCpGs
7–29 (contains retrotransposon sequences)	FM985968	4 (255 bp)	39 of 196 (20%)	38 of 40 (95%)
Phosphatase 2a gene fragment	FM985961	10 (245 bp), 11 (240 bp), 12 (219 bp)	238 of 1513 (16%)	184 of 197 (93%)
eIF2γ gene fragment	FN395317	10 (334 bp)	51 of 368 (14%)	40 of 40 (100%)
Histone H3 gene fragment	AY125256	17 (151 bp)	30 of 774 (4%)	18 of 203 (8%)
Σ		14616 bp	358 of 2851 (12.6%)	280 of 480 (58.3%)

**Figure 4:**
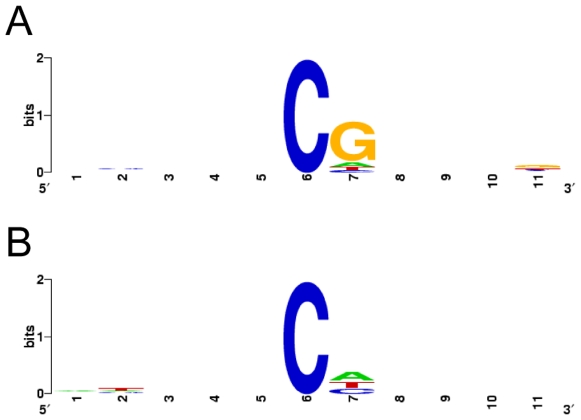
Sequence context of cytosine methylation in Medauroidea. Logos of sequence contexts were obtained from aligned 11-mer sequences in which the methylcytosine was in the sixth position. (A) All 358 11-mers were analyzed. (B) Sequences were restricted to the 77 methylcytosines in non-CpG contexts. The maximum sequence conservation per site is 2 bits, whereas 0 bit means that there is no preference for any nucleotide.

Remarkably, the fragments corresponding to the retrotransposon, the phosphatase 2a gene and the eIF2γ gene showed similarly strong signals of methylation. 15.8% of all cytosins and 94.6% of the CpG positions were methylated in these 47 clones. Clones from sense and antisense strands of the phosphatase gene showed no differences. In stark contrast, all 17 histone H3 clones were only weakly methylated (4% of all cytosins and 8% of the CpG positions). Intriguingly, the amount of CpT and CpA methylation was found to be similar as in all other bisulfite clones. Together, 3.9% of the CpT positions and 3.7% of the CpA positions are methylated (Figure 4). Thus, we detected a cytosine methylation which is nearly complete at CpG sites in two single-copy, protein-coding genes and in a transposon-derived sequence, while clones from the multi-copy histone H3 genes show a significantly lower level of CpG methylation.

### CpG depletion argues for germline methylation

As cytosine methylation was primarily found at CpG positions, we wondered if this might have been influenced dinucleotide abundances in the Medauroidea genome. A depletion of CpG dinucleotides is specifically found in organisms showing high (e.g. vertebrates) or moderate (e.g. echinoderms and tunicates) degrees of CpG methylation [13]. The most convincing explanation of a genome-wide or regional CpG depletion is the high disposition of methylcytosine to mutate via deamination to thymine [20]. If correction of the resulting T/G mispairing fails, affected CpG dinucleotides will convert in half of cases to TpG (same strand) or CpA (opposite strand). Thus, CpG depletion and a corresponding TpG and CpA enrichment are signatures of methylation occuring in the germline.

To analyze dinucleotide abundances in *M. extradentata*, we collected nuclear DNA sequences from different sources. First, we used 30 sequences originating from the elution of DNA undigested by HpaII, i.e., 23 putatively methylated sequences which were collected as described above and, in addition, 7 sequences which did not contain MspI sites and/or which were shorter than 200 bp. 15 of these sequences were repetitive as shown by Southern blot, internal repeats, and/or by BLAST analysis. Second, we cloned and sequenced fragments from 4 strongly conserved genes (eIF2γ, phosphatase 2a, chromatin assembly factor 1 and casein kinase II; accession numbers FM985958-FM985961) using degenerate primers designed for a phylogenetical project (Eisenhardt and Krauss, unpublished). Third, we added sequences of three already known nuclear genes (Histone H3, 18S rDNA, 28S rDNA; AY125256, AY121173, AY125313) [21].

In summary, we analyzed 37 DNA fragments with a cumulative length of 27256 bp (Table 2). Together, these sequences are 11% depleted of CpG and 9.5% enriched on TpG+CpA, if compared with the expected abundances derived from the frequencies of the corresponding single nucleotides. These significant deviances are based on the stronger effects of CpG methylation found in the protein-coding and the repetitive classes of sequences (Table 2). In contrast, the RNA genes show a nearly opposite bias, which might be due to strong selection concerning almost all nucleotides.

**Table 2:** Relative dinucleotide abundances (observed/expected) in different sequence classes of the *Medauroidea extradentata* genome.

Sequence class	Fragments	bp	G+C%	CpG	TpG	CpA
Protein-coding genes	5	2525	47.4	0.59***	1.28***	1.31***
Repetitive DNA	15	14788	38.4	0.76***	1.17***	1.17***
Unclassified DNA	15	5885	40.8	0.97	1.13**	1.02
28S and 18S ribosomal RNA genes	2	4058	56.5	1.10*	0.97	0.75***
∑	37	27256	42.4	0.89***	1.11***	1.08***

Stars mark significant deviances from the expected relation (1.00) according to a two-sided, exact test.

*0.05≥p>0.01.

**0.01≥p>0.001.

***0.001≥p.

To compare the extent of methylation in Medauroidea and other metazoans, we used the strong conservation of all 5 analyzed coding gene fragments and collected orthologous fragments from genome project data (Table 3). Significant overrepresentations of TpG and CpA and significant underrepresentations of CpG were found for 3 of the analyzed insects (for *Medauroidea extradentata*, the pea aphid *Acyrthosiphon pisum* and the honey bee *Apis mellifera*). All three species show a mainly CpG-oriented cytosine methylation [18], [22]. The strongest hypermutation bias of the compared genes was found in Medauroidea, which is even slightly stronger than in the corresponding human and murine genes. Therefore, mutational pressure by CpG methylation has similarly strongly influenced some coding gene sequences in Medauroidea as in vertebrates.

**Table 3:** CpG, TpG and CpA dinucleotide abundances (observed/expected) in different metazoan genes.

Organism	CpG	TpG	CpA
Danio	0.53***	1.37***	1.36***
Nematostella	0.56***	1.33***	1.25**
**Medauroidea**	**0.59** ***	**1.28** ***	**1.31** ***
Homo	0.60***	1.22**	1.16*
Mus	0.62***	1.29***	1.26**
Strongylocentrotus	0.63***	1.11	1.27***
Ciona	0.66***	1.23**	1.20**
Apis	0.72*	1.22**	1.17*
Acyrthosiphon	0.75**	1.33***	1.19*
Pediculus	0.79*	1.12	1.17*
Nasonia	0.82*	1.10	1.18*
Drosophila	0.92	1.34***	1.15*
Daphnia	0.94	1.09	1.08
Aedes	1.03	1.17*	1.16*
Bombyx	1.05	1.05	1.00
Caenorhabditis	1.12	1.00	0.98
Anopheles	1.15*	1.15	1.06
Tribolium	1.19*	0.99	1.00

Orthologous fragments of 5 strongly conserved, protein-coding genes were used. The species are ordered according to CpG depletion. The lengths of the analyzed sequences lie between 1846 and 3989 bp. Stars mark significant deviances from the expected relation (1.00) according to a two-sided, exact test.

*0.05≥p>0.01.

**0.01≥p>0.001.

***0.001≥p.

## Discussion

Insects show much less and differently distributed DNA methylation than other metazoans [2]. This is consistent with the finding that in metazoans the amount of DNA methylation is negatively correlated with population sizes and positively with the amount of cell turnover [8]. Walking sticks (Phasmatodea) possess, unlike other insects, low population sizes and high cell turnovers during development. Thus, we analyzed the occurrence and distribution of methylcytosine in the walking stick *Medauroidea extradentata* to evaluate whether they show, conclusively, stronger DNA methylation than other insects.

By comparing differential digestions, we demonstrated a genome-wide, mostly CpG-specific methylation in Medauroidea and in the related walking stick *Sipyloidea sipylus*. Prominent, undigested bands combined with smears of relatively low molecular weight as obtained in the HpaII lane from Medauroidea and Sipyloidea DNA were not found for aphids, mealy bugs, butterflies or Drosophila, but for example in digestions made from Ciona DNA [23]. The methylcytosine-specific enzyme McrBC exhibits a very similar restriction pattern for Medauroidea DNA, which also supports regional clustering of methylated and unmethylated DNA. We suggest, therefore, that walking sticks have a clustered, mosaic-type DNA methylation pattern similar to that of invertebrate chordates [24].

We isolated DNA fragments accumulated in the undigested part of the HpaII restriction and cloned 23 putatively methylated sequences that did not contain any recognizable fragments of functional genes. We confirmed methylation for 10 of these fragments by hybridization and showed that 9 of these 10 reevaluated sequences are repetitive. Therefore, the majority of the isolated sequences appears to be repetitive, which was supported by different means in 14 of 23 cases. Methylcytosines inside of repetitive and transposon-like sequences indicate a possible involvement of DNA methylation in the suppression of transposition. Functional transposons of walking sticks are not yet known, so we cannot test at this stage whether transposons are silenced by cytosine methylation. This is an attractive direction for subsequent work.

Furthermore, we demonstrated by bisulfite sequencing that, within the independently isolated fragments of two single-copy coding genes and of one fragment of the repetitive sequences mentioned above, 15.8% of all cytosines are methylated. CpG positions inside these fragments were nearly completely methylated (94.6%). In contrast, bisulfite clones from a multi-copy gene, histone H3, showed only weak CpG methylation (8%). This should be interpreted carefully as other gene copies might be strongly methylated. Interestingly, the non-CpG methylation appeared not significantly lower than in the other analyzed fragments.

In summary, 280 out of the 358 methylcytosines were followed by a guanine downstream. Also CpT (32, 3.9%) and CpA (33, 3.7%) dinucleotides seem to get occasionally methylated, while 14 methylcytosines followed by another cytosine represent only 2.3% of all CpC positions and might be, therefore, artifacts of incomplete bisulfite conversion or PCR mutations [25]. Consistently, the MspI lane of the Southern blots revealed no evidence for or against CpC methylation within 5′-CCGG-3′ sites in Medauroidea (Figure 3).

A similar quantitative relationship of dominant methylation of CpG (90%), weak methylation of CpA (2.4%) and CpT (0.7%) and no methylation of CpC (0%) dinucleotides was found in murine embryonic stem cells [26]. In contrast, Drosophila shows mainly methylation in a CpT or CpA context [27]. In the mealy bug *Planococcus lilacinus*, methylcytosine appears to occur nearly independently of the identity of the 3′ nucleotide [28], [29]. Thus, the sequence context of Medauroidea DNA methylation seems to be more similar to that of vertebrates than to that of mealy bugs or Drosophila. In vertebrates, both CpG and non-CpG methylation is dependent on Dnmt1 and/or Dnmt3a/b activity [26], [30], [31], while methylation is catalyzed exclusively by Dnmt2 in Drosophila [26]. Therefore, it will be interesting to see in the future if CpG and non-CpG methylation of Medauroidea differ in their enzymatic origin. The similar level of CpG and non-CpG methylation specifically in the histone H3 gene fragment argues in favor of a division of labor between different enzymes.

Motivated by the otherwise strong preference for CpG methylation, we evaluated the depletion of CpG and the enrichment of TpG and CpA dinucleotides caused by CpG hypermutation in Medauroidea sequences. We demonstrated that in evolutionary conserved coding genes, Medauroidea appears to be equally strongly methylated at CpG positions as vertebrates (Table 3). Interestingly, in repetitive sequences this signature is somewhat weaker (Table 2), distinct from the situation in vertebrates. Simmen [20] reports a depletion of CpG in whole mammalian genomes (dominated by repetitive sequences) to about 20% of the expected amount. While we detected methylated repetitive sequences in Medauroidea, here the amount of methylation seems to be higher within conserved coding genes if estimated according to the CpG hypermutation effect. Such a counterintuitive preferential methylation of the bodies of active genes was suggested to inhibit the initiation of transcription inside of genes [32] and was first reported for the aphid *Myzus persicae*, where DNA methylation is positively associated with the activity of amplified esterase gene copies [33]. Similarly, in Apis DNA methylation was also found primarily within genes, not at their 5′ or 3′ ends [18]. This accumulation of CpG methylation within active transcription units is not limited to insects. In humans, especially bodies of highly active genes are strongly methylated [34], [35], Suzuki et al. [36] identified DNA methylation preferentially within genes of *Ciona intestinalis*, and also in Arabidopsis active gene bodies are significantly stronger methylated than their 5′ or 3′ ends [37]. Thus, preferential methylation of gene bodies seems to be a general phenomenon in animals and plants.

In conclusion, both suppression of transposon activation and suppression of disturbing initiations of transcription inside of genes could be functions of DNA methylation that correspond to the detected distribution of methylcytosine in Medauroidea. In concordance with a quantitative shift of distribution, its function within coding genes probably has become more prevalent during the evolution of walking sticks as compared to other insects. A role of cytosine methylation for inactivation of transposons might have been maintained only in some insects such as Drosophila [11] and possibly Medauroidea, but not in others such as aphids and coccids [22], [38]. Thus, Medauroidea confirms our initial expectation that animals with low effective population sizes and high amounts of cell turnover should frequently methylate cytosines as vertebrates do. In walking sticks, DNA methylation may play, therefore, an important role for the regulation of gene activity during development [8]. This could be proved by analysis of promoter methylation of differentially expressed candidate genes.

## Materials and Methods

### Insect specimens used

*Apis mellifera* (honey bee) was trapped in the vicinity of Leipzig (Saxony, Germany). For *Drosophila melanogaste*r, a wild type strain from the laboratory was used. *Bombyx mori* (silk worm) was used from commercial stocks. Parthenogenetically reproducing strains of the walking sticks *Sipyloidea sipylus* and *Medauroidea extradentata* were reared on blackberry leaves. Before DNA isolation, the complete intestinal tract was removed to avoid contamination with plant and bacterial DNA.

### Digestions, cloning and Southern blot

DNA was isolated by standard protocols. Digestion using MspI, HpaII, MboI, Bsp143I (all from Fermentas) and McrBC (New England Biolabs) has been performed according to the conditions required by the supplier at 37°C for 16 h using 1 U/µg DNA at least three times independently using the same DNA preparations for all enzymes. Applying the programs Object Image and Graphic Converter, a two-color-plot was made from the intensity of ethidium bromide fluorescence of MspI/HpaII digested probes running side by side at a 0.8% agarose gel. For cloning of methylated DNA fragments, the band of highest molecular weight in the HpaII lane was excised from a 1.0% low melting agarose gel, heated 10 min at 70°C and supplemented immediately with Bsp143I and restriction buffer for overnight digestion. The resulting fragments were isolated using glass milk, cloned in a pBSII vector (Stratagene) linearized with BamHI and sequenced. Sequencing was performed on ABI 3100 equipment using BigDye Sequencing Chemistry (ABI). For sequence analyses, MacVector 7.2 (Accelrys) was used. Southern blots were made using 20 µg DNA and the Vaku-Blot system (Biometra). Probes were generated by PCR from pBSII-cloned insert sequences, tagged with alkalic phosphatase and, applying materials and protocols of the Gene Image AlkPhos Direct Labeling and Detection system (Amersham Biosciences), used for hybridization and detection via luminescence.

### Bisulfite sequencing

Genomic DNA of *M. extradentata* was treated with sodium bisulfite using the EZ DNA Methylation Gold Kit (Zymo Research). The amplification of the anti-sense-strang-specific subfragment of the repetitive DNA fragment 7–29 was done applying the primer pair Bac7-29-B1 (5′-GTTTTTRCCACAACAARC-3′) and Bac-7-29-B3 (5′-GGAYTAAYYTTTATYTATYA-3′) for two successive PCRs. A control experiment at *Drosophila melanogaster* DNA, performed in parallel, resulted in a conversion of 169 out of 170 cytosines to thymine.

Sense-strand-specific subfragments of the phosphatase 2a fragment were amplified applying the primer pair Bex6512-A1 (5′-CATGGAGGATTTTTTTTATTTA-3′) and Bex6512-A2 (5′-ATCACACATAAAACCCTACAAC-3′) for first PCR, primer pairs Bex6512-A1 and Bex6512-A3 (5′-TATCATAATCTAAAAACCTTAC-3′) for the 245 bp subfragment and the primer pairs Bex6512-A2 and Bex6512-A4 (5′-ATGTTTTGAGAGATTTAATTTTG-3′) for the 240 bp subfragment to generate nested PCRs based on the first PCR product. The amplification of the anti-sense-strang-specific phosphatase 2a subfragment of 219 bp was done using the primer pair Bex6512-B1 (5′-CATAAAAAACTTTCTCCATCCA-3′) and Bex6512-B2 (5′-ATAGTAGGTTATATATGGGATTTTG-3′) for first PCR and the primer pair Bex6512-B1 and Bex6512-B3 (5′-GGAATAGATAATAATAAAATGTTG-3′) for nested PCR. A 333bp fragment of the eIF2γ gene were amplified applying the primer pair Mex289-A1 (5′-AGTAGGTGGTAGTGTTTTTYG-3′) and Mex289-A2 (5′- AAAACTAACCCTAACCRTACC-3′) for first PCR, and the primer pair Mex289-A3 (5′-TTTYGAGGTGTGTTTAAGG-3′) and Mex289-A4 (5′-AACTAACCCTAACCRTACCTC-3′) for a nested PCR. A 151bp fragment of the histone H3 gene were amplified using the primer pair Mex-H3-A2 (5′-TTAATATCCTCAAACAATCCC-3′) and Mex-H3-A3 (5′- YGAYGGGAGGTAAAGTTT-3′) for first PCR, and the primer pair Mex-H3-A4 (5′-CAATCCCACCAAATAAACC-3′) and Mex-H3-A5 (5′-YGTTATAGGTTTGGTATYGTYG-3′) for a subsequent nested PCR and a reamplification of the product.

In all experiments on gene fragments, we used undegenerated nucleotide positions assuming completely converted cytosines (or guanines on the antisense strand) at non-CpG positions to ensure that only completely converted DNA fragments would be amplified. PCR fragments were cloned in pJET1.2 vectors (Fermentas) and sequenced. MacVector 7.2 (Accelrys) was used for sequence alignment of converted and unconverted fragments. Consensus sequences for cytosine methylation were generated using Weblogo 2.8.2 [39].

### Analysis of dinucleotide abundances

For analysis of dinucleotide abundances, we used MacVector 7.2 (Accelrys). Degenerate primers based on conserved amino acid sequences surrounding evolutionarily shifted intron positions (NIPs) [40] were used to amplify parts of 3 genes encoding phosphatase 2a, chromatin assembly factor 1 and casein kinase II, repectively, from Medauroidea genomic DNA via nested PCR. For phosphatase 2a, we used 6512-1 (5′-ACCCAAGTTTACGGTTTYTAYGAYGARTG-3′) and 6512-4 (5′-AATGTCCTGACCGAAIGTRTAICCIGCICC-3′) for the first PCR and 6512-2 (5′-ACGGCGCTCGTCGATGGICARATHTTYTG-3′) and 6512-3 (5′-AATACCCCAGCCCCCICKRTCRTCIGGRTC-3′) for the nested PCR. For casein kinase II, we used 8971-1 (5′-AAGAAGAAAATTAAAMGIGARATHAARAT-3′) and 8971-4 (5′-ATTATCATGGCCATGRAARAAIGGYTCYTT-3′) for the first PCR and 8971-2 (5′-GAACACGTCAATAACACIGAYTTYAARCA-3′) and 8971-3 (5′-ATTGTATTCTTGTCCIGGRTGRTARAAYTC-3′) for the nested PCR. For chromatin assembly factor 1 (CAF-1), we used 4191-1 (5′-CACAAGGATGAGATTTTYCARGTICARTGG-3′) and 4191-4 (5′-ATTCTCGGCCATTTGCCAIACYTGCATDAT-3′) for the first PCR and 4191-2 (5′-TGGGATTTGAGCAAAATHGGIGARGARCA-3′) and 4191-3 (5′-CACGGAACAAATAACCCAIGGYTCRTTIGG-3′) for the nested PCR. For the 4th gene, eIF2gamma, Trizol reagent (Invitrogen) was used to isolate total RNA. cDNA was synthesized using Hminus-M-MLV reverse transcriptase (Fermentas) and a polyT primer. RT-PCR was performed using the primers EFdeg6-2 (5′-TTTGTACCAACACCDATHARDCCICCIGG-3′) and EFdeg7 (5′-CCNGGNCAYGAYATHYTNATGGCIACIATG-3′) in the first and the primers EFdeg6-2 and EFdeg3 (5′-GARCAYTTRGCSGCYATHGARATHATG-3′) in the second round. A sequence of 450 bp was obtained, which was prolongated to 1011 bp after 5′RACE (rapid amplification of cDNA ends) using the additional primer BacEF1 (5′-GCAAAGAGGGAAACAATGC-3′). All PCR products were cloned in pJET1.2 (Fermentas) for sequencing. Orthologous gene sequences from selected metazoans were retrieved from NCBI using tBLASTn. The orthology of these candidate sequences was verified by reciprocal BLAST analysis.
